# Examination of eQTL Polymorphisms Associated with Increased Risk of Progressive Complicated Sarcoidosis in European and African Descent Subjects

**Published:** 2023-12

**Authors:** Nancy G. Casanova, Sara M. Camp, Manuel L. Gonzalez-Garay, Ken Batai, Lori Garman, Courtney G. Montgomery, Nathan Ellis, Rick Kittles, Christian Bime, Amy P. Hsu, Steven Holland, Yves A. Lussier, Jason Karnes, Nadera Sweiss, Lisa A. Maier, Laura Koth, David R. Moller, Naftali Kaminski, Joe G.N. Garcia

**Affiliations:** 1. Department of Molecular Medicine, Univeristy of Florida, Scripps, Jupiter FL, USA; 2. Center for Inflammation Science and Systems Medicine, University of Florida, Wertheim Scripps Research Institute, Jupiter FL, USA; 3. Cancer Prevention & Control, Roswell Park Comprehensive Cancer Center, Buffalo, NY, USA; 4. Oklahoma Medical Research Foundation, Oklahoma City, OK, USA; 5. University of Arizona Cancer Center, Tucson, AZ, USA; 6. Division of Health Equities, Department of Population Sciences, City of Hope, Duarte, California, USA; 7. Department of Medicine University of Arizona, Tucson, AZ, USA; 8. National Institute of Allergy and Infectious Diseases. National Institutes of Health, USA; 9. Department of Biomedical Informatics, University of Utah, Salt Lake City, UT, USA; 10. Department of Pharmacology, University of Arizona, College of Pharmacy, Tucson, AZ, USA; 11. Department of Medicine University of Illinois, Chicago, IL, USA; 12. Department of Medicine National Jewish Health, University of Colorado, Denver, CO, USA; 13. Department of Medicine University of California San Francisco, San Francisco, CA, US, USA; 14. Department of Medicine Johns Hopkins University School of Medicine, Baltimore Maryland, USA; 15. Department of Medicine Yale University School of Medicine, New Haven, CT, USA

**Keywords:** Sarcoidosis, GWAS, expression quantitative trait loci (eQTL), polymorphism, SNPs

## Abstract

**Background:**

A limited pool of SNPs are linked to the development and severity of sarcoidosis, a systemic granulomatous inflammatory disease. By integrating genome-wide association studies (GWAS) data and expression quantitative trait loci (eQTL) single nuclear polymorphisms (SNPs), we aimed to identify novel sarcoidosis SNPs potentially influencing the development of complicated sarcoidosis.

**Methods:**

A GWAS (Affymetrix 6.0) involving 209 African-American (AA) and 193 European-American (EA, 75 and 51 complicated cases respectively) and publicly-available GWAS controls (GAIN) was utilized. Annotation of multi-tissue eQTL SNPs present on the GWAS created a pool of ~46,000 eQTL SNPs examined for association with sarcoidosis risk and severity (Logistic Model, Plink). The most significant EA/AA eQTL SNPs were genotyped in a sarcoidosis validation cohort (n=1034) and cross-validated in two independent GWAS cohorts.

**Results:**

No single GWAS SNP achieved significance (p<1x10–8), however, analysis of the eQTL/GWAS SNP pool yielded 621 eQTL SNPs (p<10–4) associated with 730 genes that highlighted innate immunity, MHC Class II, and allograft rejection pathways with multiple SNPs validated in an independent sarcoidosis cohort (105 SNPs analyzed) (NOTCH4, IL27RA, BTNL2, ANXA11, HLA-DRB1). These studies confirm significant association of eQTL/GWAS SNPs in EAs and AAs with sarcoidosis risk and severity (complicated sarcoidosis) involving HLA region and innate immunity.

**Conclusion:**

Despite the challenge of deciphering the genetic basis for sarcoidosis risk/severity, these results suggest that integrated eQTL/GWAS approaches may identify novel variants/genes and support the contribution of dysregulated innate immune responses to sarcoidosis severity.

## Introduction

Sarcoidosis is a complex multisystemic disease of unknown etiology characterized by the formation of non-necrotizing granulomas in multiple organs but invariably involving lung tissues and thoracic lymph nodes [1]. Although impacting all races, ages, and genders, when compared with non-Hispanic Whites (NHWs), African-Americans (AAs) in the United States have the highest sarcoidosis prevalence, a three-fold higher lifetime risk (2.4%), and greater disease severity and extra-thoracic involvement compared to non-Hispanic Whites (NHWs) [2–4]. The clinical heterogeneity and unpredictable disease course in sarcoidosis represents a challenge to early diagnosis and prognosis as the majority of sarcoidosis patients experience spontaneous recovery (uncomplicated sarcoidosis). However, a third of the patients develop complicated sarcoidosis defined as: i) multiorgan involvement [5,6], including cardiac and nervous system involvement, and ii) progressive fibrotic lung involvement with pulmonary function deterioration. Cardiac and nervous system involvement are thought to be severe and usually require aggressive treatment [7]. Environmental and genetic susceptibility factors have been associated with sarcoidosis [3,8,9] with current concepts invoking sarcoidosis development in genetically-predisposed individuals exposed to yet unknown environmental trigger(s) that result in dysregulated immune responses [8,10,11].

We and others have previously utilized genomic expression profiling of peripheral blood mononuclear cells (PBMCs), including RNA sequencing, microRNA and protein-coding gene expression data, to sub-phenotype subjects with uncomplicated or complicated sarcoidosis [12–19]. These studies highlighted immunological dysregulation in genes/pathways associated with the T-cell receptor, CXCL9 [20] and Jak-STAT signaling pathways involving pro-fibrotic genes/proteins, including HBEG, eNAMPT and ANG-2 (angiopoietin-2), which were identified as potential biomarkers of sarcoidosis severity [21–23]. Importantly, significant progress has been made in identifying genetic variants that contribute to the development of sarcoidosis and complicated sarcoidosis [24, 25] including SNPs in the butyrophillin-like 2 gene (BTNL2), a member of the B7 costimulatory receptor family [26, 27]; and in annexin A11 (ANXA11), a calcium-dependent phospholipid-binding protein involved in cell division and vesicle trafficking [28] which can influence granuloma formation and maintenance [29]. Similarly, the D allele polymorphism in the angiotensin-converting enzyme gene (ACE) [30, 31], and NOD2, which induces constitutive activation of the transcription factor NF-kB, are associated with early-onset sarcoidosis [32]. NOTCH4 was identified as a sarcoidosis-associated risk and severity gene in AA populations, and HLA-DRB1 SNPs as risk/severity SNPs for progressive sarcoidosis in European descent subjects [4, 33] and disease susceptibility in African descent subjects [4].

Multiple linkage and association studies, including the ACCESS study interrogating genome-wide loci and familial clustering, have shown the HLA locus on the short arm of chromosome 6 to harbor variants that influence sarcoidosis risk and severity [11, 34–38]. Recently HLA-DRB1*03:01 and HLA-DQA1*05:01 alleles were associated with better prognosis in sarcoidosis [39], although MHC-related studies differ significantly among specific populations.

Our study was initially designed as a limited GWAS study of sarcoidosis cohorts comprised of European- and African-American subjects (EA, AA) with either uncomplicated or complicated sarcoidosis. Although this underpowered study failed to yield a single genome-wide significant SNP, by integrating a list of known expression quantitative trait loci (eQTL) expressed in multiple tissues with SNPs present in our GWAS platform, we successfully identified novel SNPs/genes associated with sarcoidosis susceptibility in our AA/EA sarcoidosis cohort which were represented in pathways involved in allograft rejection and MHC Class I and II genes. A number of eQTL/GWAS SNPs were successfully validated in a cohort of ~1000 genotyped sarcoidosis subjects. These studies indicate that despite the challenge of deciphering the genetic basis for sarcoidosis risk/severity, integrated eQTL/GWAS approaches may identify novel variants/genes, particularly HLA region and innate immunity variants/genes, that contribute to dysregulated innate immune responses and the risk of severe sarcoidosis.

## Materials And Methods

### Genetic analyses of European American and African American sarcoidosis subjects

A genome-wide association study (GWAS) was conducted utilizing the Affymetrix Genome-Wide Human SNP Array 6.0 Platform utilizing DNA samples and data obtained from a discovery population of 402 well-phenotyped European American and African American subjects (193 EA 209 AA) with proven sarcoidosis. DNA specimens were obtained from University of Chicago, University of Illinois at Chicago, National Jewish Health, University of California San Francisco, Johns Hopkins University, and the NHLBI repositories ACCESS and GRADS studies. A dataset from the Genetic Association Information Network (GAIN) (627 AAs, 579 EAs) was used as control (Accession phs000167.v1.p1). All studies had appropriate institutional review board approvals. The clinical characteristics of GWAS (n=402) participants are presented in Table 1. Complicated sarcoidosis was defined as presence of any indicator of parenchymal lung disease, (CT scan or radiographic Scadding stages III and IV; Forced vital capacity (FVC) <50%) and/or cardiac or neurological involvement. Due to limited information in our data sets multiorgan involvent was not assessed. The non-complicated group, was defined as sarcoidosis was limited to skin or mediastinal lymphadenopathy. DNA from a second independent cohort of well-phenotyped sarcoidosis subjects (n=1034), was utilized for MassArray-based genotyping (Agena Bioscience) targeting eQTL-SNPs in a replication study.

We excluded 123 cases due to incomplete phenotype or low call rate genotype, leaving 911 subjects included in the analysis. The characteristics of the final number of successfully genotyped subjects in the validation cohort are presented in Table 2. In limited studies, a third distinct sarcoidosis cohort was utilized for further validation of the MAssArray genotyping results. These independent GWAS data set from Adrianto I, et al. included 818 AAs and 1088 controls and two replication independent set of 442 EAs cases and 455 AAs sarcoidosis cases, sample summary details have been previously published [2].

### Sarcoidosis Discovery Cohort GWAS

High-quality DNA was genotyped on the Affymetrix^®^, 6.0 genotyping platform that included over 906,600 SNPs and more than 946,000 probes for the detection of copy number variation using GeneChip^®^ Array station with the analytical process outlined in Supplemental Figure S2. Briefly, after restriction enzyme digestion, fragments were PCR-amplified, labeled and hybridized to the array. The Birdseed genotype calling algorithm was used as implemented by the Affymetrix Power Tools (APT) software. The APT software and PLINK 1.9 were used for quality control. 402 samples passed call rates > 95%, MAF > 1% and HWE >1e-06. Controls were obtained from a publicly-available database of GWAS data from the GAIN study and provided 627 AA and 579 EA GAIN controls. Genotyping data derived from the healthy control group of the GAIN studies was matched with sarcoidosis cases according to race, age, and gender (3:1 controls/case ratio). When genotyping calling for sarcoidosis cases was performed together with the GAIN controls, overall call rate was reduced (88.7%), with Hardy-Weinberg Equilibrium p= 3.92 x <10–25. Utilization of a genotyping call rate of 0.95 yielded 402,232 SNPs in EAs and 541,309 SNPs in AAs (Supplemental Figure S2). Allele and genotype calling data was formatted to PLINK [40] to determine variant associations with sarcoidosis with each EA and AA GWAS data initially processed together and then separately applying logistic regression and additive genetic models. Variant filtering and annotation was performed adjusting for 10 PCs with VarSeq (Golden Helix, Inc. Bozeman, MT).

### Generation of an annotated eQTLs tissue-specific database

An extensive list of expression quantitative locus (eQTLs) was generated by comprehensive cis-eQTL search of the publicly-available database, Genotype-Tissue Expression (GTEx) version 7 (accession phs000424.vN.pN, October 2017) [41]. eQTLs previously identified from a total of 7 tissues frequently affected in sarcoidosis (lung, heart, arteries, spleen, skin, pancreas, brain) and blood were included. The intersection of these annotated eQTLs with the 541,309 non-intergenic SNPs present on the Affymetrix 6.0 array generate a tissue-specific pool of eQTL SNPs: lung (71,698), brain (332,322), heart (113,941), skin (90,737), arteries (212,554), spleen (34,482), pancreas (50,576) and whole blood (71,616). Elimination of redundant eQTLs yielded a pool of~46,900 SNPs that were present in both GTEX and GWAS datasets with required tissue representation in either brain, lung, heart, or blood (Figure 2).

### eQTL selection and colocalization analysis

Logistic Model Analysis (PLINK) of the extracted eQTL/GWAS dataset was next performed in each of 4 comparison groups (EA or AA cases vs controls, AA or EA complicated vs uncomplicated sarcoidosis cases) generating eQTL SNPs/group with p<10–4 and prioritized for affecting multiple genes and pathways. The resulting 2000 SNPs were evaluated for linkage disequilibrium (LD) defined as r2 >0.25 in the grouped AA or EA or at least one of the 5 EUR subpopulations or at least one of the 7 AFR subpopulations (1000 Genome). We next identified the genes influenced by these selected eQTLs resided within 25K base upstream/downstream of 5’ and 3’ UTR of the gene region defined by Genome Reference Consortium Human Build 37 (GRCh37) (https://www.ncbi.nlm.nih.gov/assembly/GCF_000001405.13/). The depiction of the steps to obtain the annotated tissue-specific eQTLs in the 4 sarcoidosis comparison groups is shown in Figure 2.

### Selection of eQTL SNPs for MassArray genotyping validation

From the 621 eQTL SNPs identified, the top 25 eQTL SNPs with the highest p value in each of the four comparison groups (AA uncomplicated, EA uncomplicated, AA complicated, EA complicated), were chosen for validation by MassArray genotyping in a validation cohort of >900 sarcoidosis subjects. In addition, the MassArray genotyping replication SNP list included SNPs selected from the 46,000 eQTL pool representing JAK-STAT pathway SNPs/genes postulated to be involved in sarcoidosis pathobiology (rs 7549445, rs6749232) [16]. Similarly, SNPs were included for validation which were previously highlighted in published studies of sarcoidosis risk and severity in AAs and EAs [2, 28, 38, 42–45]. Finally, we incorporated SNPs rs16910526 (CLEC7A), and rs5743809 (TLR6), as these were previously identified genetic studies of coccidiomycosis, a granulomatous disorder sharing a number of immunologic and histologic features with sarcoidosis [2, 16]. Thus, a total of 113 SNPs were selected for validation via MassArray genotyping (Supplementary table 1). Lastly, these SNPs were compared to independent sarcoidosis GWAS datasets that included 818 AAs cases and 1088 AAs controls and two replication-independent sets of 442 EAs and 455 AAs sarcoidosis cases [2].

### MassArray genotyping in a validation cohort

Sarcoidosis genomic DNA was extracted from peripheral blood cells using a kit from Thermo Fisher Scientific Inc., according to the manufacturer’s instructions. The Agena MassArray platform, iPLEX genotyping multiplex (Agena iPLEX assay, San Diego, CA, USA), was utilized to genotype targeted SNPs. Briefly, DNA was multiplexed end-point PCR followed by base extension, using MALDI-TOF mass spectrometry, the distinct mass of the extended primer identified each allele [46]. The Chi-square test was applied to assess the consistency of MassARRAY. Hardy-Weinberg equilibrium (HWE) was also calculated with a p value < 0.05 (two-tailed) considered as statistical significance. The genotyping results of each SNP were calculated.

### Statistical analysis

In the GWAS analysis, a Bonferroni correction for multiple testing (n=541,000 SNPs) was used as the genome-wide threshold for significance (alpha =3.50 x 10–8). For eQTL colocalization analysis, the eCAVIAR program was utilized [47] to identify variants with evidence of disease and eQTL associations. The algorithm estimates the posterior probability that the same variant is causal in both a GWAS and eQTL study while accounting for LD. eCAVIAR requires a correlation matrix among the markers included in the analysis and PLINK v1.07(40) was used to calculate the r2. Logistic regression analysis with adjustment for race, complicated status, age, sex, gender, and population stratification (the first principal component) was used in an additive genetic model. Models were performed separately for EAs and AAs subjects. Enrichment analysis was conducted in ConsensusPathDB [48]. Massarrayed genotypes were transformed into a VCF files and then analyzed using PLINK 2.0. SNPs having call rate > 95% were included in the statistical analysis Hardy–Weinberg Equilibrium (HWE) was used for assessing the quality of genotypes.

## Results

### Clinical characteristics of the sarcoidosis cohorts

This study included 402 subjects in our Discovery GWAS Cohort and 911 subjects in the Validation Cohort. The clinical and demographic variables, for the Discovery Cohort are shown in Table 1 and for the Validation Cohort in Table 2. The majority of sarcoidosis subjects were female with ages between 40–60 years old; race was self-reported, the number of EAs were higher in the validation cohort compared to EAs in the Discovery Cohort. Sarcoidosis was categorized as complicated by indicators of parenchymal lung disease (CT scan or radiographic Scadding stages III and IV; Forced vital capacity (FVC) <50%) and/or cardiac or neurological involvement. Non-complicated sarcoidosis was defined as limited to skin or the mediastinal lymphadenopathy (by CT or radiographic Stages I and II).

### GWAS analysis in sarcoidosis subjects and controls

**Figure 1.**
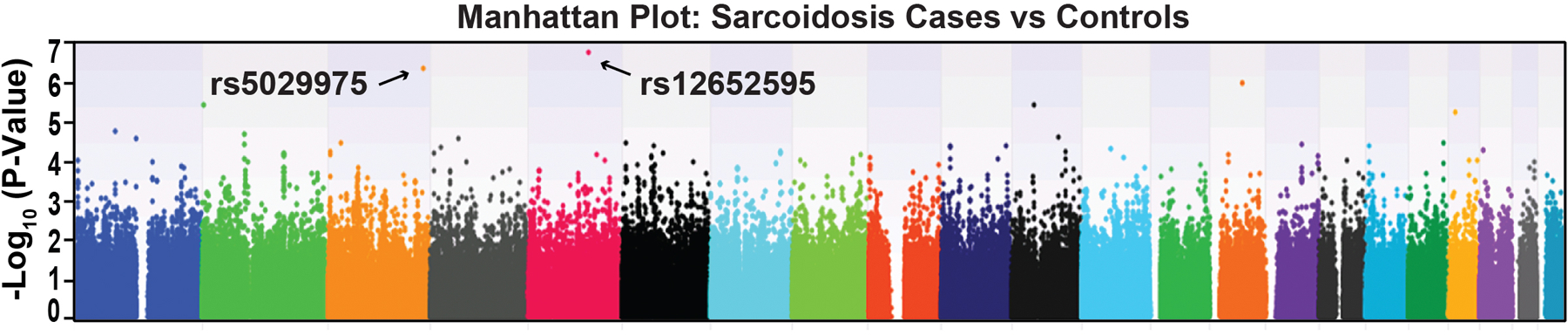
The Manhattan plot shows false positive association in EAs and AAs combines signatures. Application of logistic regression and additive genetic model GWAS analyses in the 404 sarcoidosis subjects (MAF<5%, Call rate 98%, HWE P<0.01) failed to identify any SNP which satisfied the conservative genome-wide threshold. Shown are two highest p value SNPs that did not attain statistical significance, rs5029975 in gene KNG1 and rs12652595 in LINC00992. KNG1 encodes kininogen-1, the precursor protein to high-molecular-weight kininogen (HMWK) and essential for blood coagulation and assembly of the kallikrein-kinin system. LINC00992 is a long non-coding RNAs (lncRNAs) in cellular processes during tumor progression. Neither SNP/gene has been associated with sarcoidosis.

Sarcoidosis GWAS case genotype data was merged with GAIN control genotyped data in PLINK format, utilizing the QC and genotyping parameters outlined in Supplemental Figure S2, including MAFs >0.05. For every case subject, three controls were selected (1:3 ratio) matching their gender and age group. Trend test was performed adjusting of 10 PCs with Golden Helix SVS. B. QC was performed for case data first. After merging with control data, strict QC was performed. When genotyping calling for sarcoidosis cases was performed together with the GAIN controls, overall call rate was reduced (88.7%), with Hardy-Weinberg Equilibrium p= 3.92 x 10<-25. Utilization of a genotyping call rate of 0.95 yielded 402,232 SNPs in EAs and 541,309 SNPs in AAs (Supplemental Figure S2). The application of logistic regression and additive genetic model GWAS analyses (PLINK) of sarcoidosis subjects versus GAIN controls, however, failed to identify a single SNP which satisfied the conservative genome-wide threshold of Bonferroni correction (p<3.50 x 10–8) and likewise failed to identify a single significant SNP in EA complicated vs non-complicated sarcoidosis subjects or AAs complicated vs non-complicated sarcoidosis subjects as depicted in the Manhattan plot shown in Figure 1. Two highest p value SNPs were rs5029975 in gene KNG1 and rs12652595 in gene LINC00992, however, these did not attain statistical significance.

### Integration of GWAS and eQTL SNPs for identification of race-specific sarcoidosis risk variants

**Figure 2.**
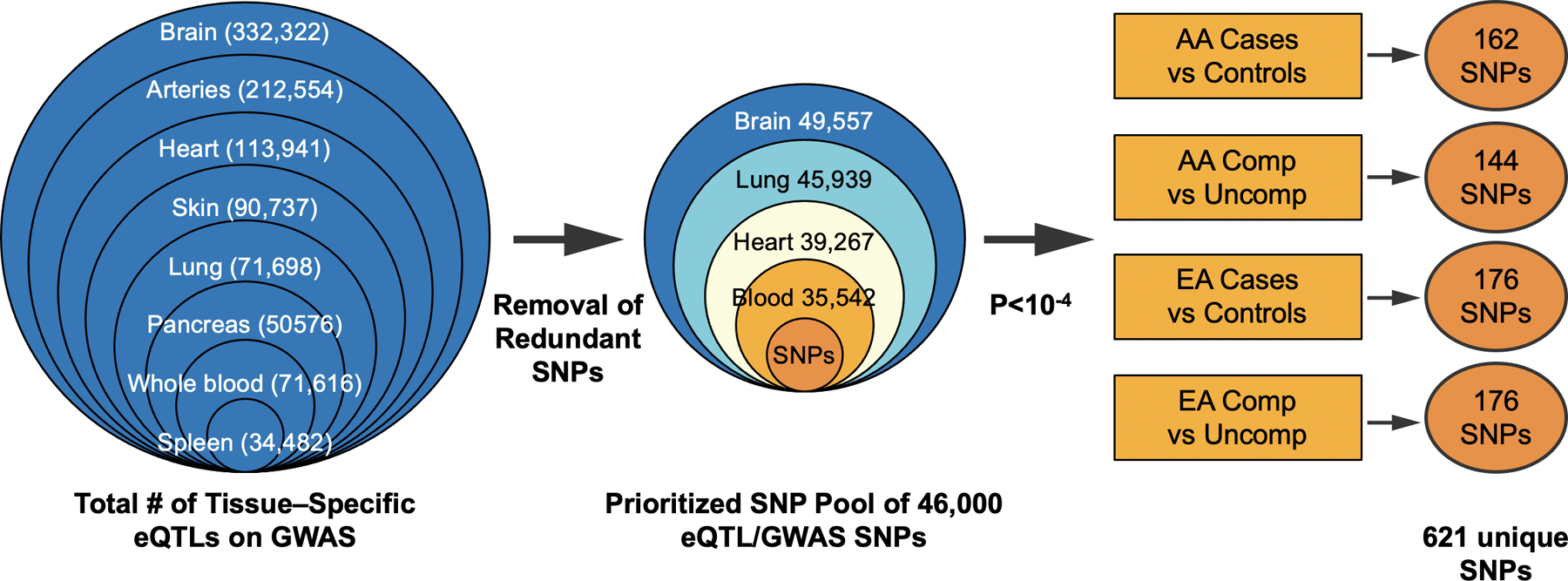
Expression quantitative locus (eQTL) selection and colocalization analysis. Logistic Model of the extracted SNPs from GWAS dataset was performed in each of 4 comparison groups (ED/AD cases vs controls, AD/ED complicated sarcoidosis cases vs uncomplicated) and then intersected with eQTLs from GTEx 7. from different tissues tissues (lung, brain, heart, skin, arteries, spleen, pancreas, and whole blood), excluding duplicates and prioritizing lung, brain, heart and blood tissues and categorized by race and complicated status according to p value (p< -E4).

**Figure 3.**
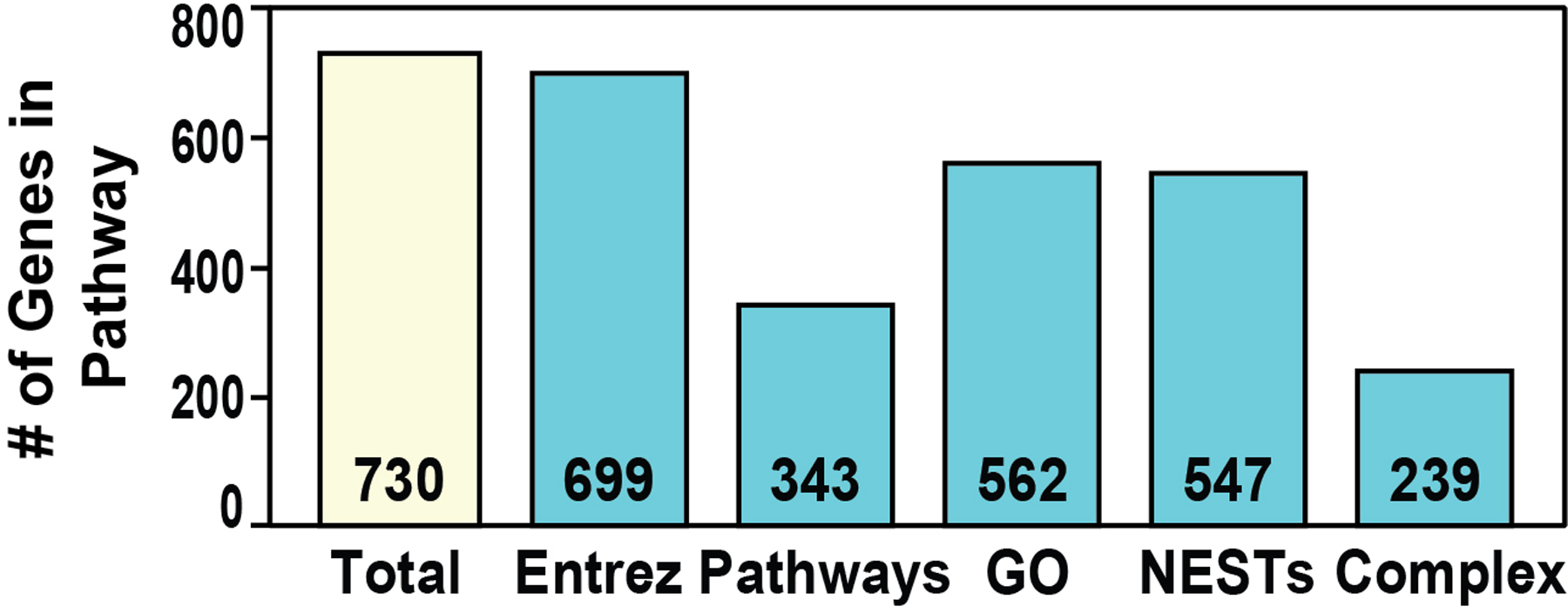
Enrichment analysis of the eQTL-associated genes revealed a total of 343 genes (49.1%) represented in at least 1 pathway (KEGG, Reactome) and 562 genes (80%) enriched in GO terms.

**Figure 4.**
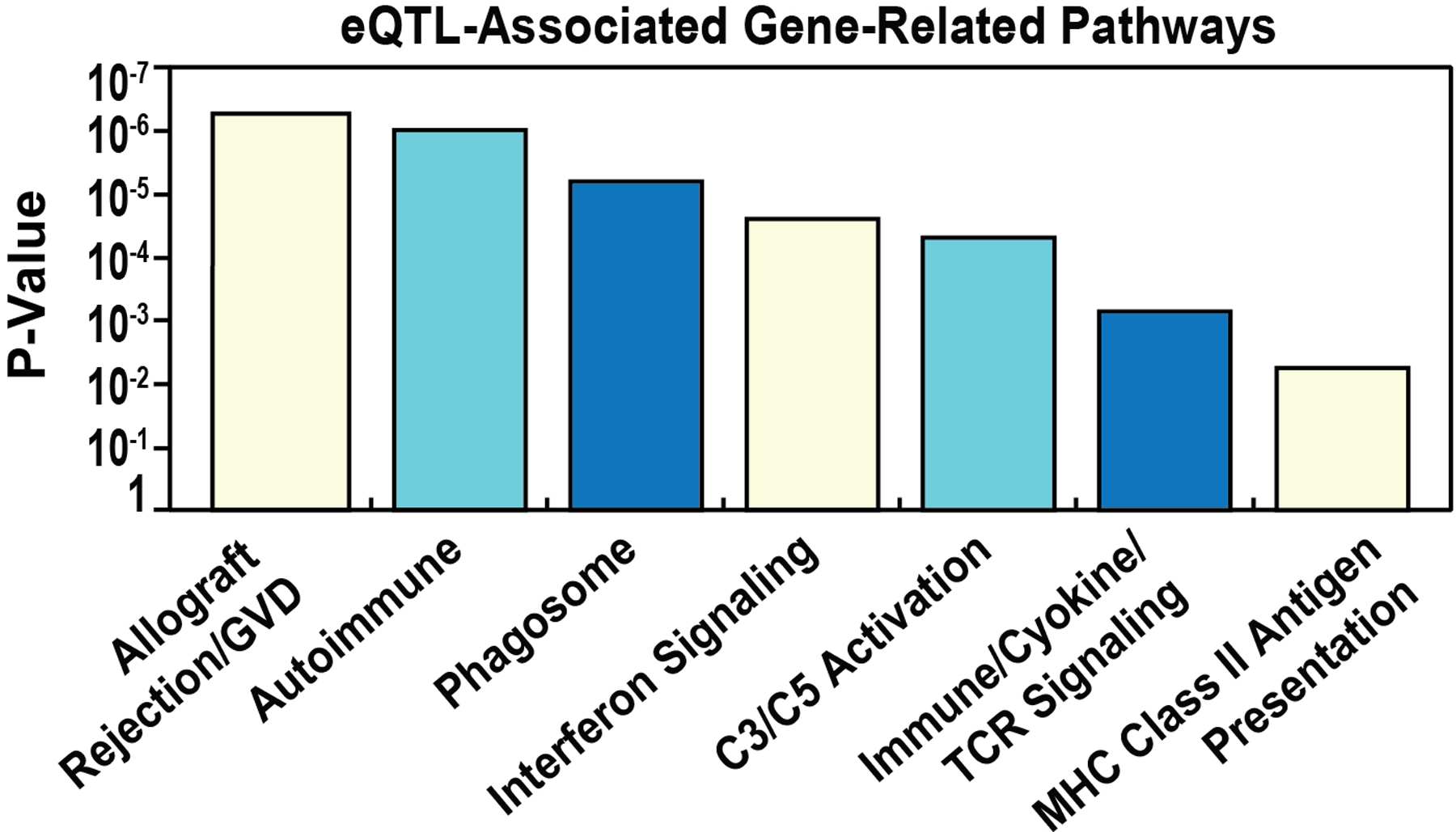
Pathway analysis of sarcoidosis vs controls. A total of 343 of the 730 genes (49.1%) were represented in at least 1 Pathway. The top most significant eQTL/GWAS SNP-related pathways include: Allograft Rejection, Graft vs Host Disease, Autoimmune Phagosome, Interferon signaling, Activation of C3 and C5, Immune system/Cytokine and TCR signaling and MHC related pathways.

From the pool of 46,965 annotated eQTL SNPs common to the Affymetrix GWAS 6.0 platform (GTEX/GWAS) and prioritized for representation in sarcoidosis tissues (lung, brain, heart, blood) (Figure 2), we next filtered for eQTL/GWAS SNPs with a p value of <10–4 within each of the 4 phenotypic categories. This revealed 162 eQTL SNPs in the AA sarcoidosis vs GAIN controls analysis, 176 eQTL SNPs in EAs vs GAIN controls, 144 eQTL SNPs in AAs with complicated sarcoidosis and 176 eQTL SNPs in EAs with complicated sarcoidosis yielding a combined total of 657, of those 621 unique eQTL SNPs (p<10–4) (Figure 2). We next generated a list of 730 unique genes known to be linked to the 621 eQTL SNPs which were found to be variably represented in various gene ontology and pathway databases (Figure 3 and 4).

### Enrichment analyses of sarcoidosis eQTLs

Enrichment analysis of the 730 gene set revealed that a total of 343 genes (49.1%) were represented in at least 1 Pathway (KEGG, Reactome) and 562 genes (80%) enriched in GO terms (Figure 3). The most significant eQTL/GWAS SNP-identified pathways are depicted in Figure 4 and include: Allograft Rejection, Graft vs Host Disease, Autoimmune, Phagosome, Interferon Signaling, Activation of C3 and C5, Immune System/Cytokine and T-Cell Receptor Signaling pathways.

**Figure 5.**
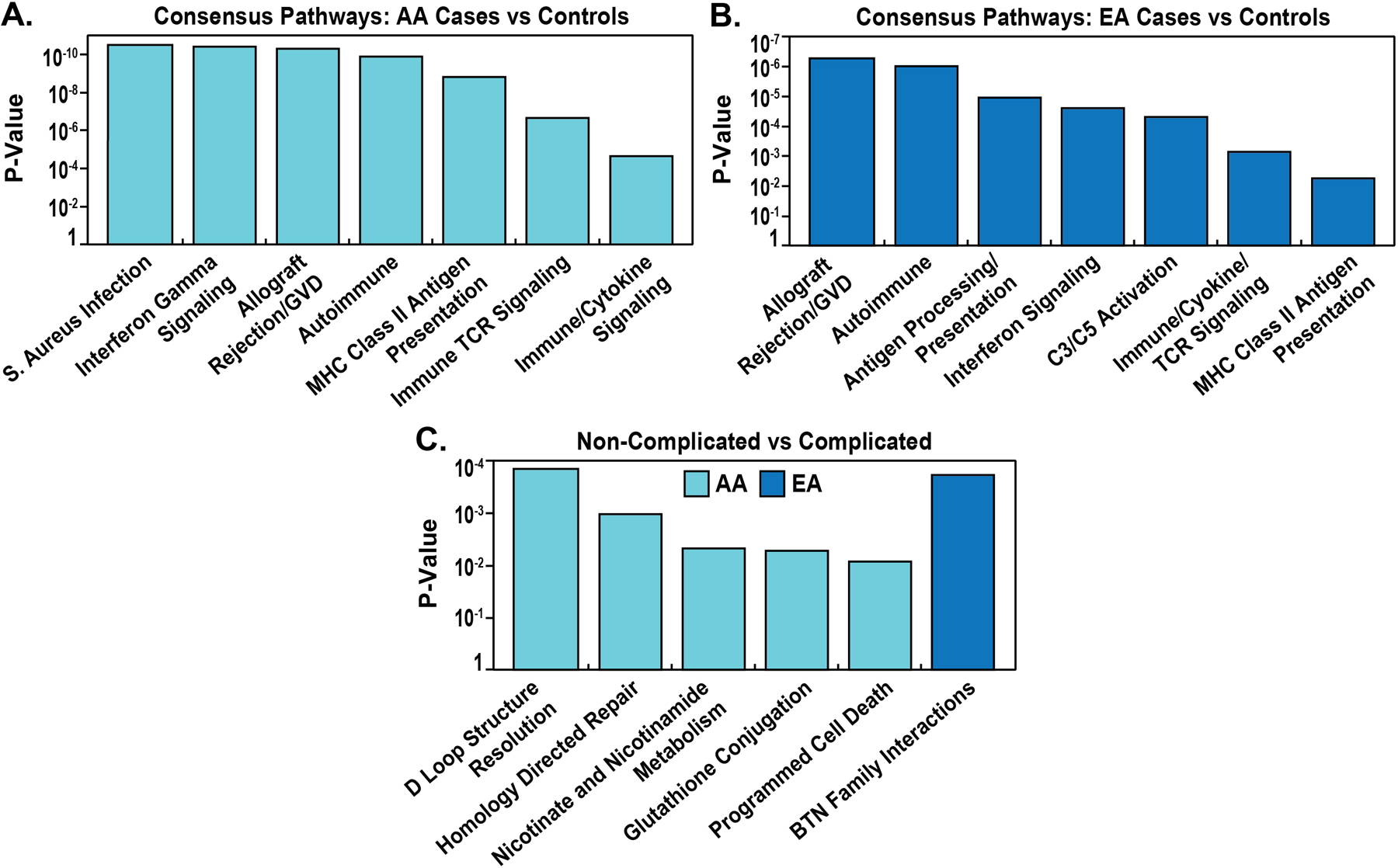
Pathway enrichment analysis of eQTL/GWAS-derived genes in AAs and EAs by complicated-and non-complicated sarcoidosis.

We next analyzed the biologic pathways derived from eQTL/GWAS SNP-linked genes specific for each of the 4 comparison groups. The eQTL-regulated gene pathways in AA cases vs GAIN controls also yielded allograft rejection, as well as Interferon Gamma Signaling, Staphylococcus aureus Infection, Graft vs Host, Autoimmune, Translocation of ZAP-70 (a surface membrane protein expressed in T cells and natural killer cells) [49] and MHC class II Antigen Presentation (Figure 5A). Interestingly, interrogation of genes involved in these pathways demonstrated very strong overlap in complement-related genes (C2, C4A, C4B) and representation by HLA genes (HLA-DMA, HLA-DOB, HLA-C, HLA-DQA1, HLA-DQA2, HLA-DQB1, HLA-DQB2, HLA-DRB1, HLA-DRB5, HLA-B). Similarly, comparison of eQTL/GWAS-linked gene pathways in EA cases vs GAIN controls also highlighted C3 and C5 complement and coagulation pathway activation, allograft rejection, autoimmune, interferon signaling and antigen presentation and Class II MHC pathways (Figure 5B).

We next compared eQTL/GWAS-regulated gene pathways in EA and AA complicated vs. uncomplicated cases revealing an absence of HLA gene-related enrichment, a significant deviation from the comparisons with controls. In contrast, the most significant EA pathways were in Butyrophylin family interactions, mismatch repair and endosomal autophagy (Figure 5C). The BTN3A2 and BTN3A3 genes in the butyrophylin family interactions pathway are known sarcoidosis candidate genes [2, 26, 43]. eQTL/GWAS-regulated gene pathways comparisons in AA complicated vs uncomplicated cases revealed purine nucleotide metabolism (ENTPD6, ADK4/5/6) and directed repair pathways (MRE11, RTEL1, EME2) (Figure 5C).

### Confirmation of eQTL/GWAS SNPs in a sarcoidosis validation cohort: high representation of the HLA region

We next sought to validate data derived from our eQTL/GWAS Discovery Cohort and from each of the four EA/AA comparison groups (complicated vs uncomplicated). A total of 113 eQTL/GWAS SNPs were selected, with genotyping data successfully generated for 103 of 113 SNPs included, with 54 SNPs called over 99.5% success rate, 467 of the 1034 (45.2%) samples in the validation cohort, genotyped at a success rate of 100% of valid SNPs, calculated out of 103 SNPs (assays with call rates > 80%). Results analysis revealed 31/103 eQTL/GWAS SNPs (27%) exhibited an eQTL p-value of 10–15 or greater, with 13 eQTL/GWAS SNPs identified to be highly significantly associated with risk of sarcoidosis (p <0.005) (Table 3). Supplemental Figure S3 shows the scatter plots with the mass intensity of each allele of those 13 SNPs. Filtering in each group was based upon p-values on the MassArray assay and the number of genes contained in eQTL pathways (Supplementary table 1). These re-sults showed that the most significant eQTL/GWAS SNPs re-sided within HLA region genes on chromosome 6 (rs2596509, rs443532, rs502771), with rs502771 in close proximity to HLA-DRB1 and NOTCH 4, a significant validated SNP reported to be linked to sarcoidosis risk in AAs (p =1.5 x 10–4). In addition to HLA gene-related SNPs, our list of validated SNPs included in-flammation-related genes such as IL27RA, RAB23, C100RF67, OS9, CCDC88B, and NOTCH4 genes, previously identified as associated with sarcoidosis risk. Only rs1442533 achieved statis-tical significance in the complicated EA subgroup (p= 3.28 x 10–3). No SNP was found to be significant in the AA complicated subphenotype analysis (Table 3).

### eQTL/GWAS SNPs compared to independent sarcoidosis GWAS datasets

Finally, we compared our 103 validated eQTL/GWAS SNPs to independent sarcoidosis GWAS datasets that included a Discovery cohort of 818 AAs and 1088 controls, an independent set of 442 EAs cases and 455 AAs sarcoidosis cases (Replication Set) [2]. These analyses confirmed five QTL/GWAS SNPs validated by Massarray that were also significantly associated with sarcoidosis in either the EA, AA discovery sets or the AA replication set (Table 4). These eQTL/GWAS SNPs included SNPs present in the AA sets (MICA-rs11753208; POL-R1B-rs11677050) and in EA cohorts (LINC00174-rs7787110; HCP5-rs3094228; RPS6KB2-rs1476792) (Table 4). In contrast to MassArray genotyping study results, the rs1476792 SNP (p < 0.0098) in the EA GWAS dataset was significant in AAs RPS6KB2/PTPRCAP -rs1476792 (p<0.007) (Table 4).

## Discussion

Prior sarcoidosis GWAS studies have successfully yielded important insights into the genetic basis for sarcoidosis risk and severity [50, 51], a significant feat given the requirement for large sample sizes and sufficient power to survive Bonferroni correction. Somewhat predictably, the analyses of our under-powered GWAS highlighted several SNPs which approached, but did not meet, genome-wide significance. Interestingly, while not significant in our analysis, several SNPs identified (Figure 1) reside within genes which exhibit biologic plausibility for an association with sarcoidosis severity and susceptibility including PTPRR, a MAPK phosphorylation regulator, down-regulated gene in lung fibrosis and IPF [52] and whose expression we have shown to be prominently dysregulated in PBMCs from sarcoidosis subjects [53]. Additional GWAS-identified SNPs included SNPs residing in ZFHX3, which encodes a transcription factor implicated in the development of inflammation and fibrosis [54], and in FAM19A5, encoding a biologic target in treatment of fibrosis [55]. Similarly, rs6748088 in FAM117B has been associated to sarcoidosis risk [27]. The HURP-generated protein promotes epithelial-to-mesenchymal transition which is recognized as involved in organ fibrosis in lung and neoplasia [56]. Nevertheless, the lack of a significant GWAS signal reduces the reliability of these SNPs as genetic predictors of sarcoidosis risk. As a result, we chose to pivot to an approach which leverages the increasing awareness of eQTL influences on disease risk/severity through regulatory impact on gene expression [47]. As a result, we integrated the readily available catalogs of eQTL SNPs with our GWAS platform as an approach designed to enhance the capacity for an underpowered GWAS studies to identify genomic regions and affected genes/pathways that associate with sarcoidosis risk and severity.

A total of 621 sarcoidosis-associated SNPs were identified via an eQTL/GWAS filtering approach utilizing our AA and EA GWAS dataset with comparison to GAIN GWAS controls. We found these eQTL/GWAS SNPs to influence 730 genes involved in pathways germane to sarcoidosis pathobiology including allograft rejection, graft vs host disease, and autoimmunity (Type I Diabetes Mellitus, Rheumatoid Arthritis, Inflammatory Bowel Disease), and major histocompatibility complex (MHC) Class II antigen presentation. The GO ontology database also highlighted MHC Class II protein/complex and T-cell receptor activity. Over 50% of the pathway candidates were represented within the 730 gene pool.

The HLA locus on the short arm of chromosome 6 encodes MHC Class II genes/proteins heavily involved in allograft rejection, antigen processing and antigen presentation and has been associated with sarcoidosis risk in both black and white populations [4, 11, 33, 57]. Candidate gene studies and GWAS studies in European- and African-descent subjects have indicated the HLA region to harbor genetic risk loci, highlighted by SNPs with significant association with sarcoidosis susceptibility within HLA-C, HLA-B, HLA-DRA, HLA-DRB5, HLA-DRB1, HLA-DPB1, HLA-DQA1, and HLA-DQB1 [37, 43, 57, 58]. However, the exact influence on risk/severity appears to be race-specific. For example, the DRB1 (*1101) allele is associated with increased risk of sarcoidosis in ADs and EDs [35], whereas DRB1 (*0101), DQB1 (*0501), and DQA1 (*0101) alleles were associated with reduced risk of sarcoidosis in diverse ED populations (UK, Netherlands), and in Japanese subjects [11, 43, 59, 60]. Our eQTL/GWAS results implicating MHC Class I and II genes are quite consistent with prior candidate gene/GWAS sarcoidosis studies [60] as we identified MHC Class I genes HLA-B and HLA-C to be well represented in EAs (compared to controls) and HLA-B primarily in AAs. MHC Class II genes represented in both EAs and AAs included HLA-DRB1 and HLA-DQA2 whereas in our study only AAs exhibited eQTL/GWAS SNPs in HLA-DMA, HLA-DOB, HLA-DQA1, HLA-DRB5, and HLA-DQB2. A recent study utilizing a similar eQTL/GWAS approach (GTEx database) but restricted to ED-only sarcoidosis subjects, identified significant sarcoidosis-associated SNPs near HLA-DQA1, an HLA allele also identified by our eQTL/GWAS approach [51]. This same study identified HLA-DRB9 and HLA-DRB5 as associated with sarcoidosis risk. Although we failed to find HLA-DRB5 as a risk locus in EDs, it was present in AAs in our study. Interestingly, the rs1964995 SNP located between HLA-DRB9 and HLA-DRB5, a risk SNP only in AAs in our study, was protective in a Swedish cohort with non-Lofgren sarcoidosis GWAS [47], but increased the risk of rheumatoid arthritis in ADs, but not in EDs [48]. Another SNP near HLA-DQA1 (rs2187668) is significantly associated with Lofgren’s syndrome [47] and HLA-DRB1*04:01 with ocular sarcoidosis in EDs [61]. The DRB1*1501 allele, in high linkage disequilibrium with DQB1*0602, is associated with increased risk of severe pulmonary sarcoidosis in EDs [20].

As a rigorous validation strategy, we assembled a list of SNPs selected from the top performing eQTLs in each of the 4 comparison groups and performed MassArray genotyping in an ~1000 subject validation sarcoidosis cohort. These results showed that 31/105 eQTL/GWAS SNPs (30%) exhibited an eQTL p -value < 10–15. The list of validated SNPs included rs502771 selected after comparison of ADs with sarcoidosis to GAIN controls residing in the HLA chromosome 6p21.3 region harboring HLA-DQA and HLA-DRB5, as well as rs9268362 and rs3094228 identified in EDs and rs502771 and rs7746922 in ADs with sarcoidosis affecting expression of HLA-DRB6 and NOTCH4, a candidate gene in the NOTCH family of T-cell immune response that regulates the activation of Toll-like receptors in macrophages, known to drive chronicity of lung inflammation [62, 63]. NOTCH4 expression is downregulated in sarcoidosis granulomas [2, 4, 11, 23, 35] and expression of the NOTCH receptor ligand is increased in cutaneous sarcoidosis granulomas [42]. A rare SNP in NOTCH4 (rs715299, MAF<0.00003) is associated with sarcoidosis in ADs and EDs independently from others in the MHC region [2].

Our eQTL/GWAS analysis also identified rs2393915 in EAs with complicated sarcoidosis associated to BTN3A2, BTN3A3 and BTN2A3P, MHC I-associated genes; other BTNL2 polymorphisms (rs2076530, rs206530), have been previously reported associated to sarcoidosis susceptibility [26, 43, 64–69]. Additional SNPS (rs6922431, rs2596509, rs11753208, rs11758964 and rs11753208) were identified as associated to MICA, which encodes the highly polymorphic major histocompatability complex class I chain-related protein A.

In addition to the HLA-related eQTL/GWAS SNPs identified, our MassArray validation studies confirmed 13 of eQTL/GWAS SNPs that were significantly associated with risk of sarcoidosis (p <0.005), including validated SNPs influencing inflammation-related genes such as IL27RA, RAB23, PSORS1C1, CTNNA1, MICA, and NOTH4. The majority of these SNPs were validated in AAs, with rs7549445, the top ranked SNP by p value, related to JAK1 gene, a Janus kinase, key component of the interleukin-6 (IL-6)/JAK1/STAT3 immune and inflammation response, supporting the importance of the JAK-STAT pathway in sarcoidosis [16, 70]. The rs1476792 SNP was also validated in ADs and is associated to RPS6KB2 (ribosomal protein), previously implicated in idiopathic pulmonary fibrosis and lung radiotoxicity via regulation of the mTOR signaling pathway [71, 72] and was dysregulated in World Trade Center responders with associated sarcoidosis [73]. Our MassArray validation study failed to identify eQTL/GWAS SNPs in HLA genes that were involved in sarcoidosis severity in AAs, however we identified, rs1442533, related to CNTLN (Centlein, centriole stabilator) associated with complicated sarcoidosis in EDs. Finally, our MassArray-targeted SNPs were also cross validated in independent GWAS datasets. This comparison identified five SNPs (rs11753208, rs11677050, rs7787110, rs3094228, rs1476792) of the thirteen eQTL/GWAS SNPs validated in our MassArray genotyping studies that were also present in the independent GWAS sarcoidosis datasets.

There are several limitations of the current study with the most glaring limitation being the small sample size of participants in the Discovery cohort undergoing GWAS interrogation. We also acknowledge the limitation related to using self-reported ancestry for the analysis. However, unlike many other genetic studies that include only individuals of European descent, our study design included sarcoidosis subjects of African descent (52%). Second, our study population was a heterogeneous population of sarcoidosis subjects manifesting multiple phenotypes thereby reducing study power for identify SNPs driving risk and/or severity. Nevertheless, our results indicate success in identifying HLA-associations that have been linked to other sarcoidosis phenotypes in previous studies such as DRB1*1101, *1501 [4, 35]. We believe our integration of eQTL/GWAS SNPs have allowed at least partial mitigation of this limitation. By utilizing the validation cohort of ~1000 sarcoidosis subjects and a mixture of different sarcoidosis phenotypes, we did not restrict enrollment to severe pulmonary sarcoidosis or other specific phenotypes. Future GWAS studies would benefit from focusing on different sarcoidosis phenotypes or those with specific organ involvement to explore genetic drivers of sarcoidosis phenotype manifestations, including neurologic sarcoidosis, cardiac sarcoidosis, or specific types of pulmonary sarcoidosis.

In summary, our findings indicate that combining a robust eQTL pool and SNPs present on our GWAS platform, and implementing multilayered replication studies and adding downstream work such as gene-set enrichment and pathway analysis, provides a useful strategy to address underpowered GWAS issues. Our results provide additional evidence that underscore HLA alleles as important functional contributors to risk of sarcoidosis disease development and progression. These eQTL/GWAS data showed that these loci act as cis-regulators of genes within pathways that have been previously identified as dysregulated in complicated sarcoidosis: allograft rejection, graft vs host, autoimmunity and innate immunity. These findings support the current understanding of disease pathogenesis as a product of the gene environment interaction and suggest that previously unknown factors in the HLA region may be regulating this response. Future studies integrating the eQTL mapping with variants discovered by GWAS may be informative for discovering genes and pathways disrupted in sarcoidosis and influencing severity of other fibrotic pulmonary disorders.

## Supplementary Material

Supplementary Table 1

Supplemental Figure S1

Supplemental figure S2

Supplemental Figure S3

## Figures and Tables

**Table 1.** Clinical characteristics of the sarcoidosis GWAS Discovery Cohort

	AD (n=209)	ED (n=193)
**Gender (n,%)**		
Female	143, 68	122, 63
Male	66, 32	71, 37
**Age (n,%)**		
< 39 years old	81, 39	82, 43
40–60 years old	113, 54	95, 49
>61 years old	13, 6.2	14, 7
**Non-complicated sarcoidosis (n,%)**	134, 64	141, 73
**Complicated sarcoidosis (n,%)**	75, 36	51, 26

**Table 2.** Clinical characteristics of the sarcoidosis MassArray Validation Cohort

	AD (n=323)	ED (n=588)
**Gender (n,%)**		
Female	227, 70	323, 55
Male	96, 28	265, 43
**Age (n,%)**		
< 39 years old	101, 31	140, 24
40–60 years old	192, 54	355, 60
>61 years old	26, 8	87, 15
**Non-complicated sarcoidosis (n,%)**	218, 67	398, 68
**Complicated sarcoidosis (n,%)**	105, 32	190, 32

**Table 3.** SNPs Validated via MassArray Genotyping in a Replication Cohort (each category compared to the original GWAS subgroups).

eQTL SNP	Position	GWAS P-value	Validation analysis	Masarray P-value	Genes
rs7248735	chr19:14019403	2.70E-03	AD and ED case vs controls	4.90E-03	IL27RA
rs10044736	chr5:138985120	5.46E-04	AD case vs control	8.32E-03	CTNNA1 SIL1
rs11677881	chr2:113760287	5.13E-04	AD case vs control	3.07E-03	DDX11L2 FAM138B PGM5P4 PGM5P4-AS1 RABL2A RPL23AP7 WASH2P
rs1476792	chr11:67428766	3.78E-03	AD case vs control	6.97E-03	PTPRCAP RPS6KB2
rs17787966	chr11:47048874	8.11E-04	AD case vs control	7.29E-03	ACP2 ARHGAP1 C11orf49 C1QTNF4 DDB2 LRP4 LRP4-AS1 PACSIN3
rs2054517	chr12:75597666	2.09E-04	AD case vs control	2.65E-04	GLIPR1 GLIPR1L2 KRR1 LOC105369844
rs2596509	chr6:31350636	9.82E-04	AD case vs control	6.59E-03	ATP6VIG2 CCHCR1 HLA-B HLA-S MICA PSORS1C1 PSORS1C2 STK19B VARS2 ZBTB12
rs443532	chr17:8884178	4.95E-03	AD case vs control	6.91E-03	PIK3R5
rs502771	chr6:32611193	1.52E-04	AD case vs control	1.50E-04	C4A C4B CLIC1 CYP21A1P CYP21A2 HLA-DMA HLA-DQA1 HLA-DQB1 HLA-DQB1-AS1 HLADQB2 HLADRB1 HLA-DRB5 HLA-DRB9 NOTCH4 PSMB9
rs6779819	chr3:50681914	8.00E-04	ED case vs control	3.28E-03	C3orf18 DOCK3 HEMK1 LINC02019 MAPKAPK3 TEX264
rs7219	chr17:75319287	2.53E-03	ED case vs control	3.28E-03	NUP85 MRPS7 GRB2 ANXA5 ILKAP
rs1442533	chr9:17379148	3.69E-04	ED complicated vs non-complicated	1.49E-03	CNTLN
rs7549445	chr1:65054080	1.02E-03	AD case vs control	3.21E-05	JAK1 RP11–182I10.3

**Table 4.** Independent GWAs data sets validation

CHR	BP	rsID	Genes	ORAA_Disc	PAA_Disc	ORAA_Rep	PAA_Rep	PAA_Meta	OREA	PEA
6	31005432	rs11753208	MICA	1.205	**4.60E-02**	8.66E-01	2.98E-01	3.18E-01	1.22E+00	1.17E-01
2	1.13E+08	rs11677050	POLR1B	1.258	**7.97E-03**	7.60E-01	**2.14E-02**	4.30E-01	1.02E+00	7.71E-01
7	23138474	**rs7787110**	LINC00174	1.025	6.77E-01	8.69E-01	1.42E-01	5.98E-01	1.19E+00	**2.50E-02**
6	31429927	rs3094228	HCP5	0.9457	8.89E-01	1.07E+00	5.53E-01	8.14E-01	1.34E+00	**1.24E-03**
11	67196237	**rs1476792**	RPSKB2	1.066	8.17E-01	9.76E-01	8.44E-01	9.43E-01	8.16E-01	**9.77E-03**

ORAA_Disc: Odds Ratio for this SNP in the AA discovery set. PAA_Disc: P-value for the SNP in the AA discovery set. ORAA_Rep: Odds Ratio for this SNP in the AA replication set. PAA_Rep: P-value for the SNP in the AA replication set. PAA_Meta: P-value from the meta-analysis of the AA discovery and the AA replication set. OREA: Odds Ratio for this SNP in the EA set. PEA: P-value for the SNP in the EA set

## Abbreviations

GWAS: genome wide association studies
eQTL: expression quantitative trait loci
SNPs: small nuclear polymorphisms
AA: African American
EA: European American
AD: Affricant Descent
ED: European Descent
NHWs: non-Hispanic Whites
HWE: Hardy–Weinberg Equilibrium
