# Supplementary material for: Examination of eQTL Polymorphisms Associated with Increased Risk of Progressive Complicated Sarcoidosis in European and African Descent Subjects
Source: Eur J Respir Med. Author manuscript; Available in PMC 2024 Feb 22. (PMC10883688)
Supplement: Supplementary Table 1 [file NIHMS1958308-supplement-Supplementary_Table_1.pdf]

Supplementary Table 1. List of Selected SNPs for Genotyping in Sarcoidosis Validation Cohort (eQTL SNP by eQTL p value and genes affected)

Group	dbSNP	Chr	p-value	eqtlPval	Gene symbols
African American Complicated vs No Complicated	rs7787110	7	9.37E-04	2.08E-68	AK3P3; GPNMB; KLHL7; KLHL7-AS1; NUPL2
	rs113413	22	9.24E-04	9.80E-62	DDT; DDTL; GSTT2; GSTT2B; MIF; MIF-AS1
	rs628728	11	1.25E-03	2.64E-33	GPR83; MRE11
	rs4148974	21	2.84E-04	2.49E-28	NDUFV3
	rs7129430	11	6.29E-04	2.26E-22	GAS2; SVIP
	rs10903129	1	8.29E-04	4.62E-21	RHCE; RHD; SDHDP6; TMEM50A; TMEM57
	rs2042370	1	8.80E-04	5.16E-20	AGMAT; CASP9; DNAJC16
	rs2178003	15	9.63E-04	2.86E-17	EHD4; JMJD7; LTK; MAPKBP1; NDUFAF1; RNA5SP393; RPAP1; SPTBN5; TYRO3
	rs1713421	14	7.68E-04	1.14E-15	OSGEP; PNP
	rs17773169	11	1.11E-03	2.94E-15	B3GAT3; EEF1G; EML3; INTS5; LBHD1; MTA2; ROM1; UQCC3
	rs2667684	16	4.00E-05	9.34E-10	EME2; IFT140; JPT2; TELO2
	rs4983589	14	9.49E-04	1.34E-08	JAG2; NUDT14
	rs6050679	20	6.99E-04	1.40E-08	ABHD12; ENTPD6; FAM182A; FAM182B; NINL; PYGB
	rs8112297	19	7.66E-04	5.43E-08	NUDT19; PDCD5; SLC7A9
	rs7614981	3	1.15E-03	5.81E-08	GLYCTK; GNL3; ITIH4; MUSTN1; NEK4; NT5DC2; PBRM1; TMEM110
	rs7007756	8	9.48E-04	1.61E-07	FUT10
	rs9195	7	5.34E-04	6.30E-07	DNAAF5
	rs12168	8	1.80E-04	1.73E-06	GSDMD; NAPRT; ZC3H3
	rs10138671	14	2.21E-04	2.57E-06	FUT8
	rs8124478	20	5.42E-04	1.61E-05	RTEL1; STMN3
African American Cases vs Controls	rs10044736	5	5.82E-04	2.18E-23	CTNNA1; SIL1
	rs544167	6	7.71E-04	3.40E-19	NELFE; SKIV2L
	rs2596509	6	7.22E-04	2.17E-17	ATP6V1G2; CCHCR1; HLA-B; HLA-S; MICA; PSORS1C1; PSORS1C2; STK19B; VARS2; ZBTB12
	rs11677050	2	3.28E-04	3.37E-17	CHCHD5; POLR1B; RGPD8; TTL
	rs10483120	22	2.22E-04	1.50E-16	CRYBB2P1; GRK3

	rs3132959	6	8.48E-04	8.69E-15	HLA-DQA2; HLA-DQB1; HLA-DQB1-AS1; HLA-DRB5; HLA-DRB6; HLA-DRB9
	rs11677881	2	6.46E-04	3.36E-14	DDX11L2; FAM138B; PGM5P4; PGM5P4-AS1; RABL2A; RPL23AP7; WASH2P
	rs606732	11	5.37E-04	3.80E-14	ALG8; KCTD21; KCTD21-AS1; NDUFC2
	rs2054517	12	1.97E-04	1.11E-12	GLIPR1; GLIPR1L2; KRR1
	rs502771	6	1.27E-04	1.52E-10	C4A; C4B; CLIC1; CYP21A1P; CYP21A2; HLA-DMA; HLA-DQA1; HLA-DQB1; HLA-DQB1-AS1; HLA-DQB2; HLA-DRB1; HLA-DRB5; HLA-DRB9; NOTCH4; PSMB9
	rs7746922	6	4.29E-04	2.15E-10	C2; C4A; C4B; CYP21A1P; HLA-DMA; HLA-DQA1; HLA-DRB5; MICB; NOTCH4; PSMB9; RNF5
	rs11752362	6	3.62E-04	1.70E-09	GABBR1; HCG20; HCG4; HLA-H; HLA-K; HLA-V; RPL23AP1; TRIM31; ZFP57
	rs6088792	20	3.59E-04	1.86E-08	GDF5; RPL36P4; UQCC1
	rs17787966	11	5.83E-05	2.10E-08	ACP2; ARHGAP1; C11orf49; C1QTNF4; DDB2; LRP4; LRP4-AS1; PACSIN3
	rs2113352	9	6.64E-04	9.58E-06	GAPVD1; PPP6C; PRPS1P2; RABEPK
European American Complicated vs Non-complicated	rs2839251	21	6.64E-04	6.33E-30	DIP2A; LSS; MCM3AP-AS1; PCNT; SPATC1L; YBEY
	rs9889755	17	4.86E-05	1.27E-27	ATAD5; CRLF3; LRRC37BP1; SUZ12P1; TEFM
	rs1026466	19	2.70E-04	3.01E-23	DUS3L
	rs11912715	22	2.26E-04	1.52E-20	MMP11; SLC2A11; SMARCB1; VPREB3
	rs11159087	14	4.68E-04	2.84E-20	NPC2
	rs1643650	5	3.34E-04	5.91E-20	ANKRD34B; DHFR; FAM151B; MSH3
	rs4965473	15	1.71E-04	3.27E-17	TTC23
	rs2349574	12	5.20E-04	3.59E-13	ERGIC2
	rs2853641	1	7.07E-04	1.88E-12	GLMP; PAQR6; SEMA4A; SLC25A44; SMG5
	rs1052053	1	2.23E-04	1.52E-11	GLMP; PAQR6; SEMA4A; SLC25A44; SMG5

	rs2393915	6	8.60E-04	2.43E-10	ABT1; BTN2A3P; BTN3A2; BTN3A3; GUSBP2; HMGN4; PRSS16; ZNF391
	rs12420420	11	9.00E-04	6.33E-10	IGHMBP2; MRPL21
	rs10083442	14	7.54E-04	5.37E-08	DAAM1; JKAMP; L3HYPDH
	rs9934251	16	3.94E-04	1.20E-07	CFDP1
	rs10924919	1	5.99E-04	1.36E-07	C1orf229; ZNF124; ZNF669
	rs10484431	6	7.23E-05	4.40E-07	TRIM38
	rs10197623	2	5.15E-05	1.49E-06	CARF; NBEAL1
	rs3750209	8	6.72E-04	3.08E-06	ZC3H3
	rs11658261	17	5.37E-04	1.15E-05	CCT6B
European Americans Cases vs Controls	rs17643040	6	1.28E-04	5.64E-37	TARID
	rs1862609	5	5.32E-05	1.33E-19	ERAP1
	rs979971	19	8.31E-06	2.27E-14	ACTN4; CAPN12; LGALS4; LGALS7; LGALS7B
	rs2844627	6	6.31E-05	3.31E-12	C4A; C4B; C6orf15; CCHCR1; CYP21A1P; HCG20; HCG22; HLA-B; HLA-C; MICB; NOTCH4; POU5F1; PPP1R18; PSORS1C2; RNF5; VARS2; WASF5P
	rs9915127	17	5.55E-04	5.67E-11	CDRT4
	rs9268362	6	1.38E-04	7.99E-11	FKBPL; HLA-DQA2; HLA-DRB1; HLA-DRB6; NOTCH4
	rs11753208	6	6.39E-04	9.45E-11	HCG22; HLA-C; HLA-L; MICA; PSORS1C1; PSORS1C2
	rs3094228	6	1.71E-05	3.35E-10	C4A; C4B; CCHCR1; CYP21A1P; CYP21A2; HCG22; HLA-C; MICB; NOTCH4; PPP1R18; PSORS1C1; VARS2
	rs1442533	9	2.94E-04	7.75E-09	CNTLN
	rs1564472	15	6.41E-04	1.84E-08	CSPG4P11; GOLGA2P7; GOLGA6L5P; UBE2Q2P1
	rs6779819	3	5.73E-04	3.85E-08	C3orf18; DOCK3; HEMK1; LINC02019; MAPKAPK3; TEX264
	rs11758964	6	2.55E-05	1.73E-07	ATP6V1G2; BAG6; HCG22; MICA; MICB; PSORS1C1; PSORS1C2
	rs1405184	15	3.95E-04	9.63E-07	MYO5A; MYO5C
	rs12738660	1	5.56E-05	2.17E-06	FCRLA
	rs6922431	6	5.15E-04	5.69E-05	MICA; MICB
[illegible] ido sis vs co	rs1040861	6	4.75E-03	1.13E-65	NQO2; RIPK1

	rs10849538	12	3.77E-03	5.90E-32	ATN1; C12orf57; CDCA3; DSTNP2; GNB3; LRRC23; PTPN6; RPL13P5; SPSB2
	rs3825075	11	7.73E-03	9.57E-19	BET1L; NLRP6; PSMD13; RIC8A; SIRT3
	rs10483120	22	2.22E-04	1.53E-16	CRYBB2P1; GRK3
	rs1044707	11	1.12E-02	4.69E-16	HRAS; LRRC56; PTDSS2; RNH1
	rs4448470	1	6.74E-03	9.65E-16	IFNLR1; IL22RA1; MYOM3
	rs1476792	11	4.40E-03	1.22E-12	AIP; CORO1B; DOC2GP; GSTP1; NDUFV1; NUDT8; PTPRCAP; RPS6KB2
	rs11170516	12	9.45E-03	2.31E-11	ATP5G2; PRR13; SP1
	rs11690687	2	7.88E-03	3.69E-11	ACTR1B; ANKRD36; ANKRD36B; LINC01125; TMEM131; ZAP70
	rs928831	22	9.34E-03	7.18E-11	CECR7; IL17RA
	rs502771	6	1.27E-04	1.52E-10	C4A; C4B; CLIC1; CYP21A1P; CYP21A2; HLA-DMA; HLA-DQA1; HLA-DQB1; HLA-DQB1-AS1; HLA-DQB2; HLA-DRB1; HLA-DRB5; HLA-DRB9; NOTCH4; PSMB9
	rs7746922	6	4.29E-04	2.15E-10	C2; C4A; C4B; CYP21A1P; HLA-DMA; HLA-DQA1; HLA-DRB5; MICB; NOTCH4; PSMB9; RNF5
	rs5751072	22	6.37E-03	1.61E-09	ACO2; EP300; L3MBTL2; MEI1; TEF; XPNPEP3
	rs7219	17	8.25E-03	2.64E-09	GRB2; MIF4GD; MRPS7; NUP85
	rs7549445	1	1.05E-03	5.89E-09	JAK1; LINC01359
	rs6749232	2	1.85E-02	6.48E-09	STAT4
	rs9878100	3	8.88E-03	7.42E-09	C3orf18; CISH; DOCK3; GRM2; HEMK1; LINC02019; MAPKAPK3; TEX264
	rs443532	17	3.90E-03	2.51E-08	PIK3R5
	rs7248735	19	3.29E-03	1.56E-07	IL27RA
	rs11585473	1	1.24E-02	6.43E-07	IL12RB2; SERBP1
	rs811322	3	9.21E-03	7.40E-07	FAIM; MRAS; PIK3CB
	rs9427089	1	9.53E-03	1.04E-06	IL6R
	rs6925103	6	1.03E-02	1.16E-06	MAP3K5; PEX7
	rs12442170	15	3.61E-03	1.42E-06	IGF1R; PGPEP1L
	rs3822606	5	1.24E-02	2.03E-06	CAMK2A
	rs2106430	22	1.19E-02	2.22E-06	INPP5J; LIF
	rs276310	5	4.25E-04	3.38E-06	GHR

	rs11670504	19	1.12E-02	4.38E-06	TYK2
	rs2274617	1	1.12E-02	7.09E-06	ANKRD35; LIX1L-AS1; PIAS3
	rs769044	14	1.09E-02	8.15E-06	PSMC1
	rs879576	22	5.61E-03	1.03E-05	IL17RA
	rs2893796	12	8.55E-04	1.09E-05	PRKAB1
	rs13361707	5	4.08E-03	1.41E-05	CARD6; PRKAA1
	rs1545255	2	3.95E-03	1.74E-05	ADCY3; CENPO; NCOA1
	rs6456103	6	6.48E-03	1.78E-05	RPS6KA2
	rs11167021	8	9.70E-03	2.12E-05	PTK2
	rs17641276	19	9.69E-03	2.39E-05	PAK4
	rs17796080	14	9.60E-03	3.00E-05	SOS2
