# Supplementary figures and images for: Examination of eQTL Polymorphisms Associated with Increased Risk of Progressive Complicated Sarcoidosis in European and African Descent Subjects
Source: Eur J Respir Med. Author manuscript; Available in PMC 2024 Feb 22. (PMC10883688)

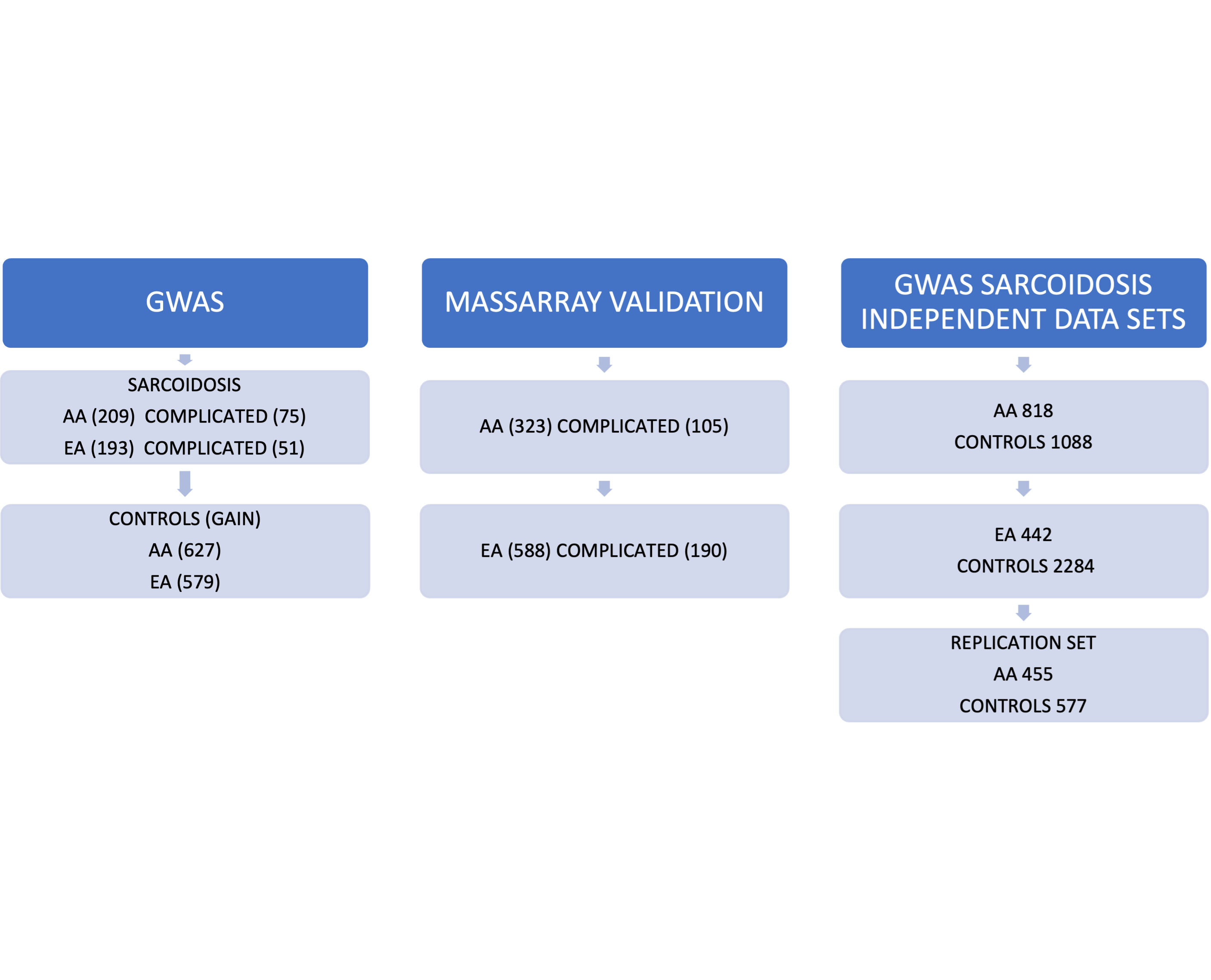

Supplement: Supplemental Figure S1 [file NIHMS1958308-supplement-Supplemental_Figure_S1.png]

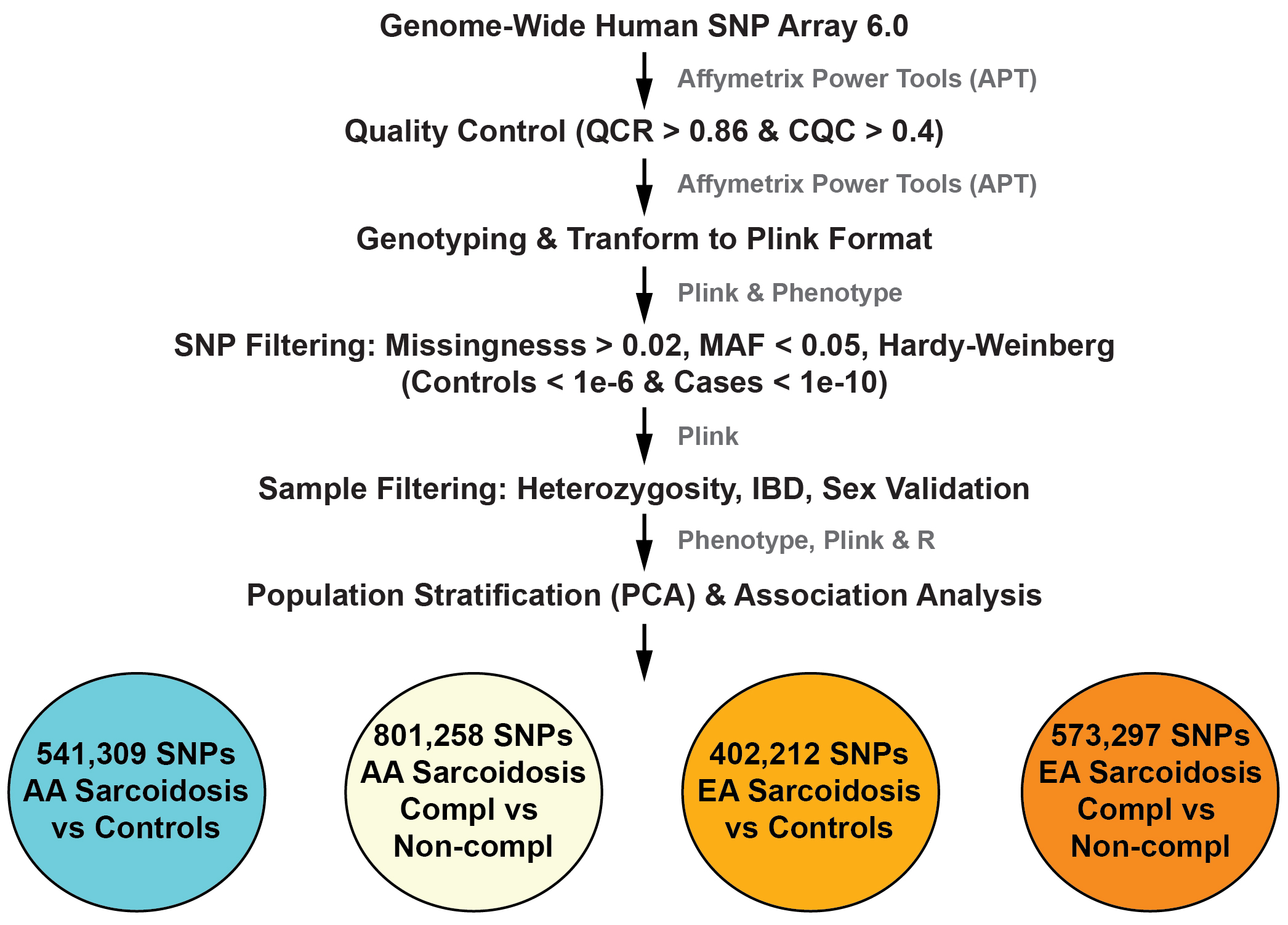

Supplement: Supplemental figure S2 [file NIHMS1958308-supplement-Supplemental_figure_S2.jpg]

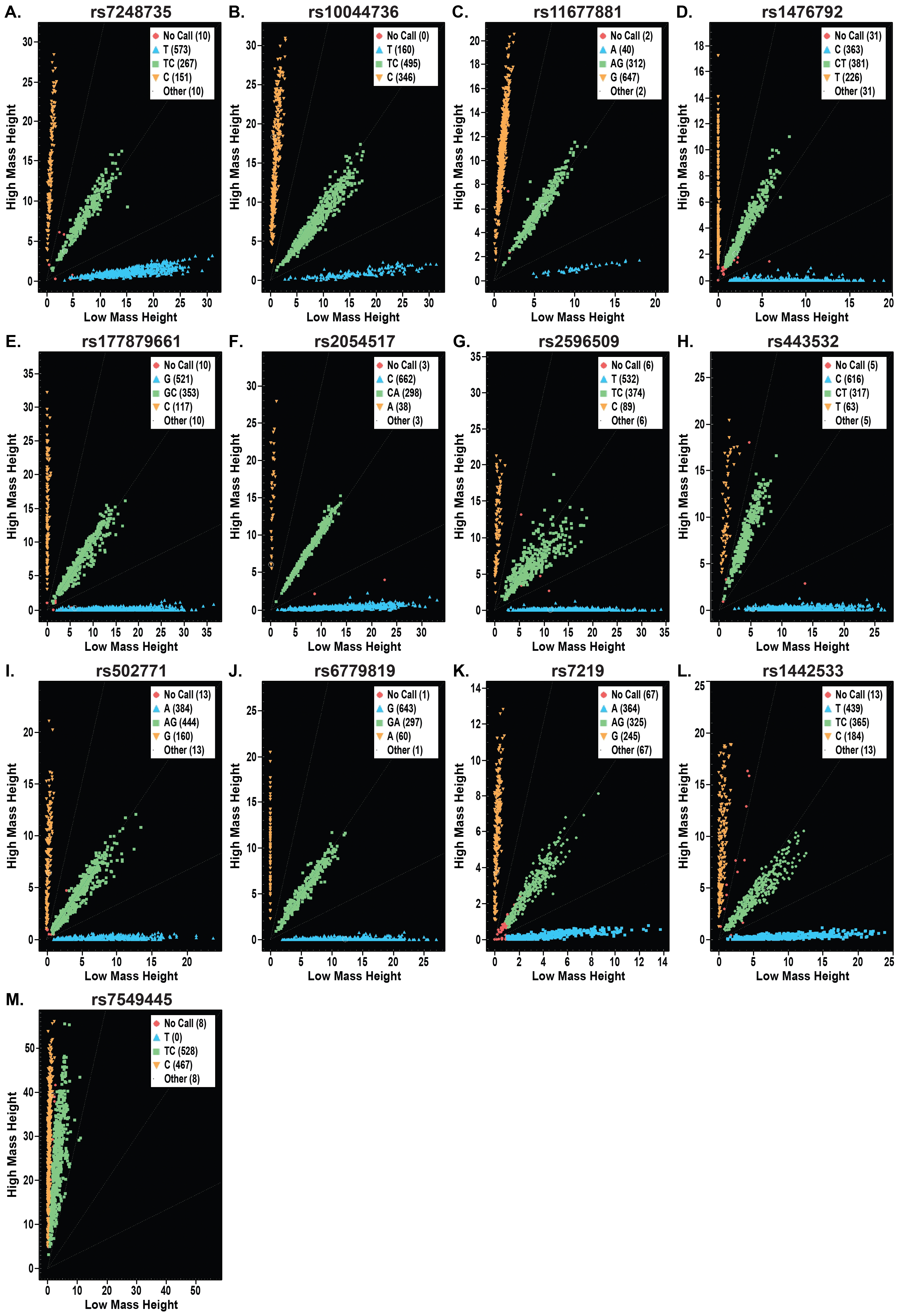

Supplement: Supplemental Figure S3 [file NIHMS1958308-supplement-Supplemental_Figure_S3.jpg]
